# Breast cancer stroma frequently recruits fetal derived cells during pregnancy

**DOI:** 10.1186/bcr1860

**Published:** 2008-02-13

**Authors:** Gil Dubernard, Sélim Aractingi, Michel Oster, Roman Rouzier, Marie-Christine Mathieu, Serge Uzan, Kiarash Khosrotehrani

**Affiliations:** 1 Université Pierre et Marie Curie, Paris VI, EA 4053. Laboratoire de Physiopathologie du développement. 184 r Fbg St Antoine 75012 Paris, France; 2 Assistance Publique-Hôpitaux de Paris, Hôpital Tenon, Service de Gynécologie, 4 r Chine 75020 Paris, France; 3 Institut Gustave Roussy, Service d'Anatomo-pathologie, 39 r Camille Desmoulins 94805 Villejuif cedex, France

## Abstract

**Introduction:**

Breast carcinomas associated with pregnancy display a high frequency of inflammatory types, multifocal lesions and lymph node metastasis. Because pregnancy results in transfer to mothers of foetal stem cells that can migrate and differentiate into various tissues, we addressed the issue of whether such cells are present in breast carcinoma associated with pregnancy.

**Methods:**

We analyzed women presenting with such tumours who were pregnant with male foetuses using fluorescence *in situ *hybridization (FISH), targeting X and Y chromosomes. The foetal cell phenotype was then determined by combining FISH and immunohistochemistry with various antibodies. Statistical analysis was performed using *t*-test or nonparametric Wilcoxon's test.

**Results:**

We found that foetal cells were present in nine out of 10 carcinomas, in contrast with none of four benign mammary lesions (*P *< 0.05). Counting foetal and maternal cells showed that the mean number of foetal cells per million maternal cells was 36 in breast cancers and 0 in control samples (*P *< 0.01). By combining FISH and immunolabelling, we found that foetal cells expressed mainly mesenchymal or, to a lesser degree, epithelial or endothelial markers, but never leucocytes.

**Conclusion:**

These findings demonstrate the frequent presence of foetal derived cells essentially in tumour stroma. Given the role played by stroma in tumour proliferation, these findings raise the issue of whether foetal cell can be targeted to influence tumour behaviour.

## Introduction

Breast cancer is among the two most commonly diagnosed cancers of pregnancy, along with cervical cancer [1]. Up to 3.8% of all breast carcinomas are associated with pregnancy [2]. These tumours account for 8% of breast cancers occurring in women younger than 45 years old and for as many as 18% of women younger than 30 years [2]. One of the most used definitions of pregnancy-associated breast carcinoma (PABC) emphasizes the fact that these tumours develop during pregnancy itself or during the first year after delivery [1,3]. Accordingly, analysis of the literature shows that PABC develops in 60% of patients during pregnancy itself and 40% of cases occurred during the year following delivery [4]. In addition, these tumours appear to exhibit peculiar profiles of evolution. Indeed, 14% to 28% of PABCs are of inflammatory types [5]. These tumours were found to be significantly larger, with more extensive lymph node involvement, as compared with tumours developing in nonpregnant women [6]. Finally, the SBR (Scarff, Bloom, Richardson; a histological index) grades of PABCs appear to be high [6,7].

Pregnancy induces the transfer of foetal cells that may persist for decades in the mother. Foetal transferred cells include differentiated cells as well as several progenitor cell types, such as haematopoietic [8] and mesenchymal stem cells [9]. These progenitors are capable of migration in maternal damaged tissues and can differentiate into various phenotypes [10]. Several groups have suggested that microchimeric foetal cells were recruited to maternal lesional tissues such as kidney, liver, or brain tissues in order to help in the repair process, and may rescue genetically deficient maternal tissues [11-13]. Similarly, we recently demonstrated the ability of foetal endothelial progenitor cells to form blood vessels in maternal inflamed skin [14].

Microchimeric cells have also been detected in tumours. Indeed, in a series of kidney transplant recipients, we and others recently reported that epidermal skin carcinoma and, moreover, Kaposi's sarcomas may arise from microchimeric donor cells [15]. Similarly, nontumoural cytokeratin-expressing foetal microchimeric cells have been identified in cervical cancer from women with a prior history of pregnancy [16].

Because PABC appears to be a frequent condition with an aggressive course, and because foetal cells can participate in tissue remodelling, we conducted the present study to analyze the presence and phenotype of foetal cells in PABCs.

## Materials and methods

### Materials

We retrospectively retrieved data from two institutions (Institut Gustave Roussy and Hôpital Tenon) between 2000 and 2005. In order to be eligible for inclusion, female patients had to fulfill the following criteria: pathologically proven ductal breast carcinoma; carcinoma diagnosed during pregnancy or during the 6 months after delivery; male child; and availability of formalin fixed paraffin-embedded specimens (biopsy, tumourectomy, or mastectomy). The study fulfilled the commitments of ethics regulations in France in terms of institutional review board approval and informed consent. Ten cases were identified; six had neoadjuvant chemotherapy and four had adjuvant chemotherapy. Control samples included benign breast tumour, such as fibroadenosis and mastosis, obtained from women pregnant with a male baby. The samples were made available to us in accordance with national and European procedures and ethical guidelines.

### Fluorescence *in situ *hybridization

Fluorescence *in situ *hybridization (FISH) was performed to detect male foetal cells in maternal breast, as was previously described by Johnson and coworkers [17], in 5 μm thick paraffin-embedded sections. We used X and Y chromosome probes labelled respectively with Cy3 (red) and FITC (green), mapping to Xp11.1q11.1 (a satellite centromeric region of the X chromosome) and to Yq12 (satellite III region of the Y chromosome; Abbott, Chicago, IL, USA).

### FISH combined with immunostaining (immuno-FISH)

In order to determine the phenotype of foetal cells detected in the sections of breast carcinomas associated with pregnancy, we combined X and Y FISH with immunostaining. This was done in similar 5 μm thick paraffin-embedded sections, as was previously described [10,18]. The antibodies were anti-CD45 (monoclonal mouse anti-human CD45, clone 2B11 + PD7/26; Dako, Carpintera, CA, USA), anti-CD34 (monoclonal mouse anti-human CD34 class II, clone QBEnd-10; Dako), anti-vimentin (monoclonal mouse anti-vimentin, clone Vim 3B4; Dako) and anti-cytokeratin (monoclonal mouse anti-human cytokeratin, clone AE1/AE3; Dako), which stain leucocytes, endothelial cells, stromal cells and epithelial cells, respectively. For the FISH combined with anti-CD34 and anti-CD45 antibody labelling, we used the Envision + peroxidase kit (Dako). For FISH combined with anti-cytokeratin and anti-vimentin labelling, we used an immunofluorescence technique with secondary goat anti-mouse antibody labelled with Texas red (Jackson immunoresearch, West Grove, PA, USA). All slides were counterstained with 4',6-diamidino-2-phenylindole.

### Scoring and statistical analysis

Sections of tissue from female pregnant patients and control sections were all analyzed for the presence of male (presumably foetal) cells. Sections were scored if more than 70% of the nuclei had at least one FISH signal. Male cells were recognized as having one X (red) and one Y (green) chromosome within an intact blue-stained nucleus. We estimated the total number of nucleated cells in each section by counting the number of nuclei in six fields that were randomly chosen (630× magnification). The size of our samples in case and control groups was identical and a total of 6 million cells were evaluated in all samples combined (range 10e^5 ^to 2 × 10e^6^).

For each case, the number of foetal cells found was reported as the total number of cells examined. The frequency of foetal cells and the proportion of tumours with foetal cells were then compared between the breast carcinomas and the control group using Student's *t*-test or nonparametric Wilcoxon's test.

## Results

### Patient characteristics

During the study period, more than 5,000 women were diagnosed with breast carcinomas in the two institutions. Sixty patients developed breast carcinoma during pregnancy or during the 6 months after delivery; only 10 had tumours that corresponded with the inclusion criteria. The clinical and histological characteristics of the patients and their breast tumours are summarized in Table 1. No patient or control individual had a history of blood transfusion, organ transplantation, or a twin sibling that could have been a confounding factor in interpreting male cell microchimerism. The mean ages of the patients and control individuals were 36 years (range 27 to 40 years) and 35 years (range 30 to 40 years), respectively. Among the malignant lesions, five were diagnosed during pregnancy at a median term of 7 months (range 2 to 7 months), and five were diagnosed postpartum at a median delay of 4 months. The four benign lesions were all diagnosed during pregnancy at a median term of 4 months (3 to 6 months). The median sizes of the malignant tumours and benign lesions were 36 mm (range 5 to 80 mm) and 28 mm (range 22 to 70 mm), respectively.

**Table 1:** Clinical and histological characteristics of the patients and breast tumours

Patients/control individuals	Time of diagnosis	Term of diagnosis (months)	Neoadjuvant chemotherapy	pTNM	Size (mm)	Nodal status	Histology	Grade	Estrogen receptors	Presence of MC cells	Number of MC cells	Number of MC cells/10e^6 ^maternal cells
Patients												
1	Pregnancy	2	Yes	T3N0	60	3N+	Ductal carcinoma	3	1	Positive	16	8
2	Pregnancy	7	Yes	T4N0	4 6	15N-/15	Ductal carcinoma	3	0	Positive	20	78
3	Postpartum	6	No	T2N0	25	11N-	Ductal carcinoma	3	0	Positive	3	7
4	Pregnancy	-	No	T3N1	30	14N+	Ductal carcinoma	3	0	Positive	23	24
5	Postpartum	4	No	T2N1	27	2N+	Ductal carcinoma	3	1	Positive	17	165
6	Postpartum	3	No	T2N1	5	24N-	Ductal carcinoma	1	1	Positive	19	29
7	Pregnancy	7	Yes	T4D N3	80	24N+	Ductal carcinoma	3	0	Positive	21	16
8	Postpartum	5	Yes	T3N1	70	6N+	Ductal carcinoma	2	1	Positive	6	12
9	Postpartum	1	Yes	T2N1	40	13N-	Ductal carcinoma	2	1	Positive	10	26
10	Pregnancy	7	Yes	T2N1	36	N+	Ductal carcinoma	3	1	Negative	0	0

Controls												

1	Pregnancy	4	-	-	70	-	Mastosis	-	-	Negative	0	-
2	Pregnancy	4	-	-	28	-	Fibroadenosis	-	-	Negative	0	-
3	Pregnancy	4	-	-	28	-	Fibroadenosis	-	-	Negative	0	-
4	Pregnancy	4	-	-	22	-	Fibroadenosis	-	-	Negative	0	-

MC, microchimeric

The analyzed specimens consisted of four tumourectomies, five mastectomies and five biopsies (four benign lesions and one carcinoma).

### Detection of foetal cells

**Figure 1:**
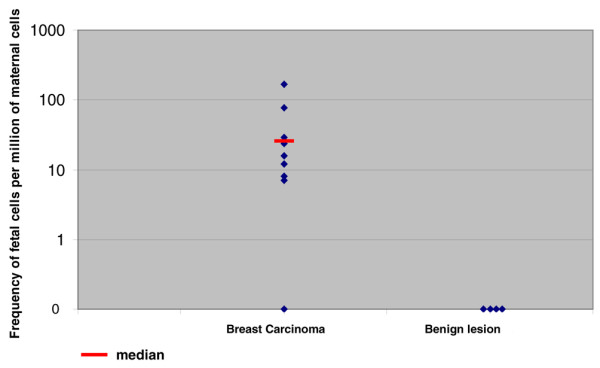
Frequency of foetal cells per million of maternal cells in breast carcinoma and in control group.

We detected X and Y positive foetal cells in maternal tumoural tissues in nine out of 10 ductal carcinomas versus none of four breast adenofibroma or mastosis samples (*P *< 0.01) (Table 2). The unique PABC that did not exhibit foetal cells was a small biopsy specimen. Surgical samples contained healthy and breast carcinoma tissue. Microchimeric cells were distributed throughout the specimens and were preferentially located in tumoural area rather than nontumoural areas. Morphologically, they were undistinguishable from the surrounding cells in terms of size and nuclear shape. Of note, the foetal cells were never grouped in clusters. We estimated the frequency of the foetal cells; we found an average of 19 foetal cells per million maternal cells (median frequency: 20 fetal/million maternal cells) versus none per million maternal cells in control individuals (*P *< 0.01; Figure 1). In addition, we compared the level of faetal cells according to patient features. There were fewer foetal cells in patients who had received neoadjuvant chemotherapy than in those who were not treated (23 versus 56 foetal cells/million maternal cells), but the difference did not reach statistical significance. No relationship were established between timing of surgery (during versus after pregnancy), hormone receptor status, lymph node invasion and number of foetal cells.

**Table 2:** Level of foetal cells observed in breast carcinoma and in control group

Patients/control individuals	Presence of MC cells	Number of MC cells	Number of MC cells/10e^6 ^maternal cells	Cytokeratin	CD34	CD45	Vimentin
Patients							
1	Positive	16	8	4	0	0	-
2	Positive	20	78	0	0	0	-
3	Positive	3	7	0	0	0	-
4	Positive	23	24	1	0	0	0
5	Positive	17	165	1	0	0	-
6	Positive	19	29	4	0	0	-
7	Positive	21	16	0	0	0	2
8	Positive	6	12	1	0	0	-
9	Positive	10	26	1	1	0	-
10	Negative	0	0	-	-	-	-

Controls							

1	Negative	0	-				
2	Negative	0	-				
3	Negative	0	-				
4	Negative	0	-				

MC, microchimeric.

### Phenotype of foetal cells

**Figure 2:**
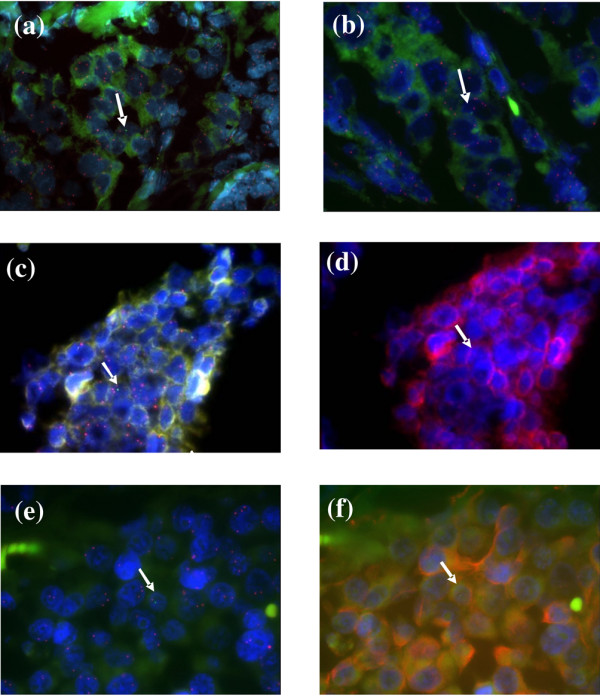
Photomicrographs showing male cells with X (red) and Y (green) chromosome signals within breast carcinoma after fluorescence *in situ *hybridization (FISH). **(a,b) **Male cells after FISH (arrows). Original magnifications: 630× for panel a and 1,000× for panel b. **(c,d) **A cluster of cytokeratin-positive cells: panel c shows male cells after FISH in a cluster of maternal cells (arrow), and panel d shows cytokeratin-positive male cell after FISH and immunolabelling (arrow). Original magnification: 1,000× for both panels. **(e,f) **A cluster of vimentin-positive cells: panel e shows a male cell after FISH in a cluster of maternal cells (arrow), and panel f shows a vimentin-positive male cell after immunolabelling (arrow). Original magnification: 1,000× for both panels.

We next determined the phenotype of foetal cells in maternal breast tumours according to four different markers. We used CD45 to identify leucocytes, CD34 for endothelial cells, and cytokeratin and vimentin for epithelium and stroma, respectively (Table 2). In the 135 foetal cells that were identified and evaluated individually for phenotype, vimentin expression and cytokeratin expression were identified in 22% (2/9 microchimeric cells) and 16% (12/74 microchimeric cells), respectively (Figure 2). CD34 expression was found in only one out of 20 analyzed foetal cells (5%). Foetal cells never expressed CD45.

## Discussion

This study was conducted to determine whether there is an interaction between a tumour type that is known to develop during pregnancy and foetal cells, which can migrate and differentiate in various maternal tissues. Our findings show that among women bearing a child, only those with breast carcinoma had foetal cells in their mammary tissue. Although the number of specimens analyzed was low, significant differences from benign control samples were identified; moreover, breast carcinomas nearly always had foetal cells within tumoural areas.

PABC is usually defined as occurring during pregnancy, lactation and up to 1 year after delivery [1,3]. In this study, in order to improve the stringency of detecting PABC and to avoid coincident cases, we restricted our sampling to tumours occurring up to 6 months after delivery. In our view, this allowed us also to avoid other unknown factors that may interfere with foetal cell trafficking over a prolonged period and to focus on a period characterized by higher levels of circulating foetal cells. Depending on country, breast carcinomas are the first or the second most common cancer occurring in pregnant women [1]. It develops in about one in 3,000 pregnant women and therefore represents a troublesome but not infrequent disease [19]. The profile of breast cancer during pregnancy has a number of distinct features. Women with PABC have larger tumours and with lymph node invasion occurring in 56% to 89%, as compared with 38% to 54% in breast carcinomas not associated with pregnancy [3,5,6,20-25]. Similarly, women with PABC were more likely to have tumours of high histological grade, to exhibit a high level of mitosis, and to be progesterone receptor negative than matched nonpregnant control women [26,27]. However, in two studies [24,28], when patients and nonpregnant control women were matched for histological grade and tumour stage, survival rates were equivalent. Therefore, the poorer scores of PABCs may result from a more advanced stage at diagnosis, because physiological changes in the breast delay diagnosis [23,29-32]. Alternatively, these higher scores may reflect an intrinsically worse profile for these breast carcinomas.

Analysis of the precise types of infiltrating foetal cells, which were present at very low levels, proved difficult. Although our team has accumulated experience in combining FISH and immunolabelling, each antibody in various tissues required careful evaluation, with multiple preliminary trials. Because this multistep, delicate technique was applied to few cells, only 40% of total foetal cells could be identified with precision, which is in accordance with our usual findings under such circumstances. Foetal cells in the vicinity of breast carcinomas were not circulating leucocytes nor endothelial cells, but seemed mainly of mesenchymal or epithelial origin, because these expressed vimentin or cytokeratin, respectively. Because the foetal cells that we identified were isolated and not organized as clusters, they were not part of the neoplastic clone, even if they expressed antigens that can be found within the adenocarcinoma.

Pregnancy induces the transfer of foetal haematopoietic, mesenchymal and endothelial stem cells to the mothers. These may persist decades after delivery in niches such as the mother's bone marrow. The persisting foetal stem cells may interact with maternal peripheral tissues. Indeed, several human and murine studies have demonstrated that foetal derived cells were present in thyroid, intestinal liver, neuronal, glial [12] and kidney tubular cells [11]. Interestingly, most studies have demonstrated that these foetal derived cells home to lesional tissues, where they adopt the phenotype of the affected tissues [11,13,33]. Therefore, it appears that foetal cells are recruited in damaged organs, where they differentiate or fuse with host cells to adopt the phenotype of the involved tissue.

In the present study, we describe foetal derived cells expressing fibroblast, epithelial and even endothelial markers in breast carcinomas. These findings are in accordance with the above-mentioned previous studies and demonstrate that the stroma of malignant tumours developing during pregnancy nearly always recruits foetal derived cells. In mice as well as in humans, CD34 haemopoietic progenitors may develop into isolated epithelial cells without clusters in injured tissues, similar to our findings [34,35]. Mesenchymal stem cells can be recruited by cancer tissue, as was previously described [36]. More recently, we reported the observation that endothelial progenitor cells of foetal origin could form blood vessels in maternal inflammatory skin during pregnancy [14]. All of these data can therefore account for the presence of foetal fibroblasts, and endothelial and epithelial cells in breast carcinomas.

Although, Cha and coworkers [16] in cervical carcinomas in women with a prior history of pregnancy reported the presence of CD45^+ ^leucocytes in 44.4% but also cytokeratin foetal derived cells in 24.3%. The present study is the first systematic evaluation of foetal cell invasion in carcinomas developing during or shortly after pregnancy. We previously conducted a study of skin epithelial tumours in skin cancers developing after kidney transplantation, a situation similar to pregnancy because it is characterized by the presence of a small number of circulating donor cells [15]. In that context, microchimeric cells from the donors were frequently found in cutaneous malignant epithelial tumours, but they were not epithelial [15]. In a unique and peculiar case, such donor cells were even able to give rise to a skin carcinoma, a situation that we did not identify in the present study. Interestingly, Kaposi's sarcoma in kidney transplant recipients occured more frequently in donor tissue [37]. However, these tumours are not epithelial but derive partly from endothelial cells. Endothelial progenitors circulate and may therefore have been present in the transplanted kidney. Therefore, malignancies occurring during pregnancy appear to frequently recruit foetally derived cells and, in the case of breast, these are mainly cells stroma participating. Gadi and Nelson [38] recently found that detection of foetal cells occurred more frequently in peripheral blood from women without breast carcinomas than in those presenting with breast cancer. These authors therefore suggest that circulating foetal microchimerism could 'protect' women from developing breast carcinoma [38].

Pregnancy associated carcinomas are aggressive tumours. Our small series of 10 patients suggests that PABC is characterized by the presence of foetal cells in stroma. Stroma may influence tumor cell proliferation and may eventually be relevant to prognosis [39]. This observation is potentially important enough to warrant further investigation. Indeed, foetal derived stromal cells may behave in a different way from adult maternal cells, because foetal mesenchymal cells exhibit a greater degree of plasticity, grow more rapidly and exhibit longer telomers than do their adult counterparts [40]. In addition, the maternal immune system allows fetal cells bearing paternal nonshared antigens to avoid immune responses. Human leucocyte antigen (HLA)-G is among the molecules that are expressed by foetal cells to reduce maternal immune responses [41]. HLA-G abnormal expression has also been suspected of playing a role in various malignancies, including breast cancer [42]. One can therefore hypothesize that foetal cells in breast cancer tumours may influence tumour evolution. However, this question should be addressed through comparisons of foetal cell infiltration between PABC and similar tumours occurring years or decades postpartum.

## Conclusion

The presence of foetal cells in PABC is a frequent phenomenon. These cells were mostly part of the tumour stroma and could intervene in the poorer profile of these carcinomas.

## Abbreviations

FISH = fluorescence *in situ *hybridization; HLA = human leucocyte antigen; PABC = pregnancy-associated breast carcinoma.

## Competing interests

The authors declare that they have no financial competing interests (political, personal, religious, ideological, academic, intellectual, commercial, or any other) to declare in relation to this manuscript.

## Authors' contributions

SA, KK and SU contributed to study conception and design. GD, MO, RR and MCM contributed to generation of data. KK, SA, GD and RR were involved in the analysis and interpretation of data. KK, SA and GD were involved in drafting the manuscript or revising it critically for important intellectual content.
